# Factors associated with non-adherence to Artemisinin-based combination therapy (ACT) to malaria in a rural population from holoendemic region of western Kenya

**DOI:** 10.1186/1471-2334-12-143

**Published:** 2012-06-24

**Authors:** Elizabeth O Onyango, George Ayodo, Carren A Watsierah, Tom Were, Wilson Okumu, Samuel B Anyona, Evans Raballah, John M Okoth, Sussy Gumo, George O Orinda, Collins Ouma

**Affiliations:** 1 Department of Public Health, Maseno University, Maseno, Kenya; 2 University of Minnesota, Centre for Global Health Research, Kenya Medical Research Institute, Kisumu, Kenya; 3 Department of Pathology, Kenyatta University, Nairobi, Kenya; 4 Department of Medical Biochemistry, Maseno University, Maseno, Kenya; 5 Department of Biochemistry and Biotechnology, Kenyatta University, Nairobi, Kenya; 6 Department of Nursing, Masinde Muliro University of Science and Technology, Kakamega, Kenya; 7 Department of Religion, Maseno University, Maseno, Kenya; 8 Department of Biomedical Sciences and Technology, Maseno University, Maseno, Kenya

**Keywords:** ACT, *P*. *falciparum*, Malarial treatment

## Abstract

**Background:**

Over the years, reports implicate improper anti-malarial use as a major contributor of morbidity and mortality amongst millions of residents in malaria endemic areas, Kenya included. However, there are limited reports on improper use of Artemisinin-based Combination Therapy (ACT) which is a first-line drug in the treatment of malaria in Kenya. Knowing this is important for ensured sustainable cure rates and also protection against the emergence of resistant malarial parasites. We therefore investigated ACT adherence level, factors associated with non-adherence and accessibility in households (n = 297) in rural location of Southeast Alego location in Siaya County in western Kenya.

**Methods:**

ACT Adherence level was assessed with reference to the duration of treatment and number of tablets taken. Using systematic random sampling technique, a questionnaire was administered to a particular household member who had the most recent malaria episode (<2 weeks) and used ACT for cure. Parents/caretakers provided information for children aged <13 years. Key Informant Interviews (KIIs) were also conducted with healthcare providers and private dispensing chemist operators.

**Results:**

Adherence to ACT prescription remained low at 42.1% and 57.9% among individuals above 13 and less than 13 years, respectively. Stratification by demographic and socio-economic characteristics in relation to ACT adherence revealed that age (*P* = 0.011), education level (*P* < 0.01), ability to read (*P* < 0.01) and household (HH) monthly income (*P* = 0.002) significantly affected the level of ACT adherence. Consistently, logistic regression model demonstrated that low age (OR, 0.571, 95% CI, 0.360-0.905; *P* = 0.017), higher education level (OR, 0.074; 95% CI 0.017-0.322; *P* < 0.01), ability to read (OR, 0.285, 95% CI, 0.167-0.486; *P* < 0.01) and higher income (Ksh. > 9000; OR, 0.340; 95% CI, 0.167-0.694; *P* = 0.003) were associated with ACT adherence. In addition, about 52.9% of the respondents reported that ACT was not always available at the source and that drug availability (*P =* 0.020) and distance to drug source (*P* < 0.01) significantly affected accessibility.

**Conclusions:**

This study demonstrates that more than half of those who get ACT prescription do not take recommended dose and that accessibility is of concern. The findings of this study suggest a potential need to improve accessibility and also initiate programmatic interventions to encourage patient-centred care.

## Background

Malaria is still a major cause of morbidity and mortality worldwide [1] especially in developing countries such as sub-Saharan Africa. It is estimated that about 40% of the world’s population live in malariouos areas [2] with most of the malaria-related morbidity and mortality resulting from *Plasmodium falciparum* infection [3]. Furthermore, it has been demonstrated that about 90% of the mortalities occur in sub-Saharan Africa, with 85% of these malaria-related mortalities occurring in children below five years of age and in pregnant women [3]. In Kenya, malaria is estimated to affect over 11 million people each year, majority of which, are from *P. falciparum* holoendemic transmission areas [4] with about 20-45 % of hospital admissions and 25-35 % of outpatient clinic visits due to malaria infection [2].

Despite the enormous health burden placed on millions of lives each year in sub-Saharan Africa, there has been a lot of concern regarding the usage of anti-malarial drugs [5]. Prompt and effective treatment has been key in combating malaria and prevention of disease [6]. Previous studies reported that prescribing health care physicians and the patients, particularly those that rely on the government supplies, continually battle with ‘a drug is out of stock’ syndrome and these challenges lead to non-adherence to effective anti-malarial drugs [7]. Another challenge for effective intervention is non-adherence due to socio-economic, cultural, environmental factors and individual differences [8]. Non-adherence to these drugs may promote resistance by the malarial parasites to the available drugs. For example, chloroquine, a drug that was once highly effective against the parasites that cause the disease, has become compromised as drug-resistant *P. falciparum* parasites have become increasingly prevalent in the recent decades [9]. This was followed by resistance to other drugs, including sulfadoxine-pyrimethamine (SP), mefloquine, and quinine [10]. Globally, anti-malarial drug resistance undermines efforts to reduce the public health burden in areas where malaria transmission occurs [10].

Artemisinin-based Combination Therapy (ACT) is one strategy recommended to increase cure rates in malaria and to contain resistance by *P. falciparum* worldwide [2]. ACT has been recommended as a first-line drug in treatment of malaria in Kenya since 2004 [11]. At the time of its introduction, there was no proper guideline on ACT use [12] and the drug soon became a routine medication given to patients presenting at the health facilities with clinical symptoms of malaria [12]. The drug was and is currently being prescribed without laboratory confirmation for malaria, particularly in remote areas where most health facilities do not have laboratory services [12]. Among the questions surrounding this treatment prescription is the extent to which patients complete the recommended doses. With such improper usage, there is likely to be resistance to ACT by parasites in populations resident in *P. falciparum* holoendemic region such as in western Kenya. As such, it is critical to establish the extent of drug adherence and accessibility, particularly in the remote and resource-constrained areas such as in western Kenya [13].

In 1994, the Government of Kenya implemented the National Drug Policy to check on medication, prescription and promotion of rational drug use [14]. As such, any drug that was brought into the Kenyan market had to be guided by this policy. However, it is not clear if this policy has an impact on the effective treatment of ACT [12]. The current study site, a remote location within Siaya County, is situated in a *P. falciparum* holoendemic transmission area of western Kenya. Malaria transmission occurs all year round, peaking in the rainy season months of April and May [15]. The patterns of use of anti-malarials include self-prescription, sharing of drugs with friends and relatives and premature discontinuation of treatment course [16]. Due to high endemicity, the households living in these regions have adopted mechanisms for surviving constant malarial episodes rather than seek adequate healthcare. In order to inform effective treatment intervention, it is imperative to assess the adherence levels, causes of non-adherence and accessibility to ACT in this population resident in this holoendemic region of Siaya County in western Kenya.

## Methods

### Study setting

The current study was carried out in Southeast Alego location in Karemo division of Siaya County, Nyanza Province, Kenya. In 2009, the national housing and population census, recorded that Karemo division had a total population of 84,986 with an urban population of 19,611. The total area of the division is 235.1 km^2^ with a population density of approximately 350 people/km^2^[17]. Karemo division has five locations; Siaya township, North Alego, East Alego, South Alego and Southeast Alego locations. Southeast Alego location consists of four sub-locations; Masumbi, Nyang’oma Kogelo, Mur-Ng’iya and Bar-Agulu sub-locations. The area experiences two rainy seasons; the long rainy season (between April-June) and the short rainy season (between October-December). Challenges faced by the community include low farm productivity, high rates of unemployment, and most importantly, resource-constrained health facilities due to poverty. These act as the primary drawbacks in the control of infectious disease such as malaria, with the highest rates of infections being experienced immediately after the rainy seasons [17].

### Study participants

**Figure 1:**
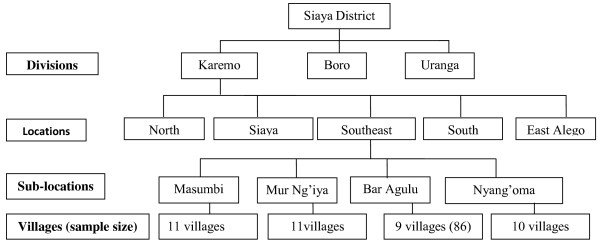
**The figure presents the different levels of clustering in the sampling frame.** Proportionate analysis was used to determine the sample size for each of the sub-locations.

In order to determine adherence level, and causes of non-adherence and accessibility of ACT in the study population at a point in time, residents living within households (n = 297) in the above described remote region of Siaya County in western Kenya, were recruited. This was a cross-sectional survey in which sampling was carried out using multi-stage and probability proportion to size sampling methods. Under these methodologies, rather than using all the elements contained in the selected clusters, the researcher randomly selected elements from each cluster. Constructing the clusters was the first stage while deciding what elements within the cluster to use was the second stage. The divisions, locations, sub-locations and the villages formed the clusters. Siaya District has three divisions namely Karemo, Boro and Uranga divisions (according to the administrative boundaries at the time of the study) (Figure 1). Karemo division has five locations namely; Siaya Township, North Alego, East Alego, South Alego and Southeast Alego was selected as study units at the division level. Southeast Alego location, which has four sub-locations namely Masumbi, Nyang’oma Kogelo, Mur Ngiya and Bar Agulu was selected at the location level. The sample size was drawn from all the four sub-locations of Southeast Alego location. The sub-locations had a total of forty two (42) villages. To ensure equal representation from each of the sub-locations and the villages, data on the number households in all the sub-locations and villages were obtained from Kenya Bureau of Statistics offices in Siaya District. The obtained data was then used in determining the proportionate sample size for each sub-location and village. This site was targeted for the study since it is one of the areas with highest malaria burden in the district. In addition, interventions such as stable supply of ACT were being implemented in this study area at the time of the study (Figure 1). These methodologies were applied since a complete list of all members of the population did not exist. This strategy was preceded by systematic random sampling in 388 households (in which a member had used an anti-malarial two weeks prior to the interview). The sample size was based on Fisher’s formula for sample size estimation [18] taking into account population size greater than 10, 000 (i.e. a total of 17,780 people were resident in the study area at the time of the study). The formula for sample size determination used the prevalence of non-adherence to anti-malarial and this was estimated at 50% since the prevalence is not known.

### Sampling design

Multi-stage and probability proportion to size methods were used to determine the sample size per sub-location in the study area. Systematic random sampling method was then used to sample out the households to be recruited as sampling unit [19,20]. Under this methodology, a member with a recent malaria episode from every third household on entering a village was recruited and interviewed. In the absence of a recent malaria case (within two weeks prior to the survey), the interviewer moved to the next household. From the 388 sampled households, a total of 297 who were either laboratory-diagnosed or had self-reported episodes, used ACT in treating malaria. The others (91) used other anti-malarial drugs which were not artemisinin-based. In the absence of laboratory diagnosis, malaria according to residents in the study area was defined by either the presence of/or mixed symptoms of fever, diarrhoea, vomiting and joint pains. However, if more than one individual in a household had experienced malaria and had used anti-malarial within the described period (i.e. two weeks), then focus was on the most current episode, while the rest were excluded. This strategy was adopted so as to capture the most recent pattern of anti-malarial drug use and to minimize errors associated with recall. In children <13 years, the parents/caregivers or a legal guardian were interviewed. Data was collected through interviews, using structured questions in the selected households. Information on the anti-malarial drugs taken, adherence and causes of non-adherence to ACT and accessibility to the drug were collected. Adherence level to ACT was then assessed by duration of treatment and number of tablets taken in relation to the age of the patient.

Key Informant Interviews (KIIs) were also conducted with Kenyan regulatory body-approved healthcare providers and dispensing chemist operators in the study area. This included 3 health care providers [1 at Ng’iya Health Centre (HC), 1 at Nyang’oma Kogelo Dispensary and 1 at Bar-Agulu Dispensary) and with 3 pharmacists (1 at Ng’iya shopping center (SC), 1 at Nyang’oma Kogelo SC and 1 at Bar-Agulu SC). Saturated sampling design was used to arrive at the number of the key informants. Through the KIIs, information on adherence to prescribed drugs, reasons for non-adherence and accessibility of ACT was collected. The study was approved by the ethical and scientific review committees at the Maseno University, Kenya and the Kenya National Council for Science and Technology Board.

### Research procedure

Quantitative data was collected from 388 households [from which those using ACT (n = 297) was obtained]. The interview was carried out using structured pre-tested questionnaire, administered to the particular family member who had malaria and used the anti-malarial drugs in the household. However, in children below <13 years, parents/caretakers or a legal guardian provided the information. Using multi-stage and systematic random sampling, households with recent malaria episodes (two weeks prior to interview date) were established. In each sub-location in the study area, a sample proportion to the total household size was sampled and interviewed. Particular emphasis was on the most recent episode of self/presumptive or laboratory diagnosed malaria in the household within the last two weeks.

Prior to the actual study, field assistants’/enumerators (n = 4) who are residents of Southeast Alego location and with a minimum of fourth form level of formal education were interviewed and recruited. The enumerators were fluent in English, Swahili and Luo languages. Training of the research assistants on survey interviewing techniques was performed for one day, followed by another day of pre-testing of survey tools and methodologies in Pap Nyadiel village, Masumbi sub-location (a neighboring region exhibiting the same characteristics as the study site). This village was then excluded during the main data collection. Based on the experiences and results of the pre-test, further re-training and refining of techniques of interviewing and modification of research tool was done. The research assistants used drug charts containing samples of commonly used anti-malarial medicines to aid recall and validate reports, remaining packages or tablets were reviewed where possible. Hospital cards were also reviewed to determine the duration of treatment. Data was collected by the researcher and field assistants. Qualitative data was also collected through KIIs with healthcare providers and chemist shop owners. Written informed consent was provided by the study participants or, where participants were children, a parent or guardian.

### Statistical analyses

Statistical analyses were performed using Statistical Packages for Social Sciences (SPSS, ver. 15.0). Descriptive analysis was then conducted to determine adherence level to ACT with reference to duration of treatment and number of tablets taken in relation to the age of the patient as recommended by the Ministry of Public Health and Sanitation and Ministry of Medical Services in Kenya (Table 1). In addition, a multivariate logistic regression analyses between the independent and dependant variables, was used to identify variables associated with adherence to ACT. The independent variables in the regression model included the following factors; demographic (age, gender, marital status, household head, and household size); socio-economic (source of income, household monthly income, education level and ability to read) and environmental (source of drugs, distance to source of drugs, and availability of anti-malarial drug at source). Chi-square analyses were used to determine proportionality. Statistical significance was assessed at *P* ≤0.050. Data collected by KIIs was organized into themes and analyzed.

**Table 1:** Dosing schedule for Artemether-Lumefantrine (Coartem)

**Weight(Kg)**	**Age in years**	**Number of tablets per dose**					
		**Day 1**		**Day 2**		**Day 3**	
		**1**^**st**^**dose**	**8 hours**	**24 hours**	**36 hours**	**48 hours**	**60 hours**
5-14	5 months ≤ 3 years	1	1	1	1	1	1
15-24	3-7 years	2	2	2	2	2	2
25-34	8-11 years	3	3	3	3	3	3
Above 34	≥ 12 years	4	4	4	4	4	4

Source: [13] National Guidelines for the Diagnosis, Treatment and Prevention of Malaria in Kenya.

## Results

### Demographic and socio-economic characteristics of the study participants

Table 2 presents the demographic and socio-economic characteristics of the study participants. A total of 388 households sampled were included in the analyses. Out of 388 households, 297 (76.5%) used ACT (Coartem/Artemether Lumefantrine) while 91 (23.5%) used other anti-malarial drugs. Overall, males were 186 (47.9%) while females were 202 (52.1%). A total of 162 (41.8%) were aged <13 years while 226 (58.90%) were ≥13 years. Majority of households consisted of 3–5 individuals [200 (52.5%)], others were >6 individuals [155 (40.0%)] and 1–2 individuals [33(8.5%)]. Households headed by husbands were 269 (69.3%) and wives, 119 (30.7%). Respondents who were married comprised of 105 (27.1%), single 79 (20.4%), widowed 30 (7.7%), while underage (<15 years and assumed to have not acquired marriageable age) were 169 (43.9%) (Table 1).

**Table 2:** Demographic and socio-economic characteristics of the study population

**Study participants**			**n = 388**		
Used ACT			297 (76.5 %)		
Did not use ACT			91(23.5 %)		
Demographic Characteristics		Number (%)	Socio-economic Characteristics		Number (%)
Age	<13 years	162 (41.8 %)	Education level	None	40 (10.3 %)
	≥13 years	226 (58.2 %)		Primary	188 (48.5 %)
Gender	Male	186 (47.9 %)		Still in school	7 (1.8 %)
	Female	202 (52.1 %)		Secondary	116 (29.9 %)
Marital status	Married	105 (27.1 %)		Post-Secondary	37 (9.5 %)
	Widowed	30 (7.7 %)	Ability to read	Able to read	265 (68.3 %)
	Separated	5 (0.9 %)		Unable to read	123 (31.7 %)
	Single	79 (20.4 %)	HHSI	Salaried employment	47 (11.9 %)
	Underage	169 (43.9 %)		Self-employed	67 (17.3 %)
HH head	Wife	119 (30.7 %)		Petty business	80 (20.6)
	Husband	269 (69.3 %)		Casual work	44 (11.3 %)
HH size	1-2	33 (8.5 %)		Farming	151 (38.9 %)
	3-5	200 (52.5 %)	HHMI	<KShs.4,500	204 (52.6 %)
	≥6	155 (40.0 %)		KShs.4,500-9,000	133 (34.3 %)
				Over KShs. 9,000	55 (13.1 %)

Legend: ACT- Artemisinin-based combined therapies, HHSI- Household source of income, HHMI- Household monthly income. From these results, majority of the respondents were ≥13 years (58.2 %), female (52.1 %) and most HH had 3–5 occupants. The respondents with primary education were 48.5 %, majority were able to read (68.3 %) written prescription, farming is the main HHSI and most HH have a monthly income of < KShs. 4,500 (52.6 %). KShs. 80 ~ 1$.

Amongst the parents/caretakers, 40 (10.3%) did not have any formal education, 188 (48.5%) had primary education, 116 (29.9%) had secondary education, 37 (9.5%) had post-secondary education, while 7 (1.8%) were still in school. A higher proportion of the respondents (68.3%) were able to read, while 31.7% were unable to read the written prescription (Table 2). Farming (38.9%) was the main source of income; others were petty business (20.6%), salaried employment (11.9%), casual work (11.3%), and self-employment (17.3%). The respondents with monthly income of below KShs. 4,500 (~ $56) were 204 (52.6%), between KShs. 4,500-9,000 ($56-112) were 133 (34.3%), while those with over KShs. 9,000 (>$ 112) were 55 (13.1%) (Table 2).

### Sources of anti-malarial drugs in the rural population

Results on the sources of anti-malarial drugs in Southeast Alego location demonstrated that a total of 77.8% of all anti-malarial drugs were from government institutions, 17.8% from nearby shops/pharmacies/chemists, 3.1% given by neighbor/ friend/ relative, and 1.3% were left-over drugs in the house (previously given in the hospital).

Out of those who used ACT (Coartem; n = 297), 278/297 (93.6%) obtained the drugs from government institutions, 14/297 (4.7%) were given the drugs by friend/neighbor/relative, 3/297 (1.0%) bought them from pharmacy/shop and 2/297 (0.7%) used left-over medicine in the house. Since the study mainly focused on ACT, distance to the source of ACT was measured based on the time taken to reach ACT source while walking. The respondents who used <30 minutes were 44.8% while 55.2% used >30 minutes.

### ACT adherence level in a rural population of western Kenya

In the context of the current study, adherence was defined as abiding by the recommended dose and period of usage of ACT. In order to assess level of adherence to ACT use, the number of tablets taken in relation to age and days of treatment were evaluated. Results revealed that most of the respondents who took ACT [215/297 (~72.0%)] had the recommended number of tablets according to their ages while others [82/297 (~28.0%)] either took less or more than the recommended number.

Duration of treatment was also assessed and results demonstrate that 161 (54.0%) took ACT within the recommended 3 days, 22 (8.0%) within <3 days while 114 (38.0%) within >3 days. When combined (duration of treatment and number of tablets taken), adherence level was found to be 140 (47.0%) for adhered and 157 (53.0%) for non-adherence.

Further analysis on adherence was conducted to determine the level of adherence with reference to demographic and socio-economic factors of the study population. General stratification of adherence to ACT and demographic factors revealed the following patterns: 57.9% of individuals <13 years and 42.1% of those >13 years of age adhered. Additional stratification of demographic factors [gender, marital status, household (HH) head, and HH size] and adherence was also conducted. Chi-square analyses revealed that age significantly differed between the ACT adherents and non-adherents (*P =* 0.011). However, gender, marital status, HH head, and HH size were comparable between the ACT adherents and non-adherents (*P* = 0.178, *P* = 0.220, *P =* 0.140, and *P =* 0.121, respectively) (Table 3)*.*

**Table 3:** Demographic factors associated with ACT adherence in Southeast Alego location

**Demographic factors**		**Adhered**	**Did not adhere**	**P-value**^**a**^	**OR**	**95 % CI**	**P-value**^**b**^
Age	<13 years	81 (57.9 %)	69 (43.9 %)	0.011	0.571	0.360-0.905	0.017
	≥13 years	59 (42.1 %)	88 (56.1 %)		1.00 (reference category)		
Gender	Male	62 (44.3 %)	79 (50.3 %)	0.178	1.274	0.807-2.013	0.229
	Female	78 (55.7 %)	78 (49.7 %)		1.00 (reference category)		
Marital status	Married	30 (21.4 %)	42 (26.1 %)	0.220	1.540	0.872-2.720	0.137
	Widowed	11 (7.9 %)	11 (7.0 %)		1.352	0.551-3.319	0.510
	Separated	1 (0.7 %)	3 (1.9 %)		3.380	0.344-33.234	0.296
	Single	18 (2.9 %)	31 (19.7 %)		1.779	0.922-3.434	0.086
	Underage	80 (57.1 %)	71 (45.2 %)		1.00 (reference category)		
HH head	Wife	34 (24.3 %)	48 (30.6 %)	0.140	1.373	0.821-2.296	0.227
	Husband	106 (75.7 %)	109 (69.4 %)		1.00 (reference category)		
HH size	1-2	5 (3.6 %)	10 (6.4 %)	0.121	2.339	0.757-7.229	0.140
	3-5	66 (47.1 %)	88 (56.1 %)		1.559	0.975-2.500	0.065
	≥6	69 (49.3 %)	58 (36.9 %)		1.00 (reference category)		

Legend: HH- Household, ACT- Artemisinin-based combined therapies. From the results, age significantly affected adherence in the study population (*P* = 0.011). ^a^Statistical significance determined by Chi-square tests. *P-*value is significant at *P ≤* 0.050*.*^b^Statistical significance determined by logistic regression tests.

In order to determine adherence level with reference to socio-economic factors, the study participants were stratified according to education level, ability to read, income per month and occupation. Chi-square analyses demonstrated that education level (*P <* 0.01), monthly income (*P =* 0.002) and ability to read (*P <* 0.01) significantly differed between adherents and non-adherents (Table 3). However, HH income source was comparable between the ACT adherents and non-adherents (*P =* 0.094) (Table 4).

**Table 4:** Socio-economic characteristics and factors associated with adherence to ACT in Southeast Alego location

**Socio-economic factors**		**Adhered**	**Did not adhere**	**P-value**^**a**^	**OR**	**95 % CI**	**P-value**^**b**^
Education level	Primary	70 (50.0 %)	73 (46.5 %)		0.074	0.017-0.322	<0.01
	Secondary	47 (33.6 %)	42 (26.8 %)		0.059	0.013-0.264	**<0.01**
	Post-Secondary	17 (12.1 %)	11 (7.0 %)	<0.01	0.038	0.008-0.195	>0.01
	Still in school	4 (2.9 %)	2 (1.3 %)				
	None	2 (1.4 %)	29 (18.5 %)		1.00 *(reference category)*		
HH inc. source	Salaried employment	23 (16.4 %)	16 (10.2 %)		0.370	0.175-0.782	0.009
	Self employed	28 (20.0 %)	21 (13.4 %)		0.444	0.224-0.878	0.020
	Petty business	29 (20.7 %)	31 (19.7 %)	0.094	0.632	0.335-1.192	0.157
	Casual worker	17 (12.1 %)	19 (12.1 %)		0.661	0.310-1.410	0.284
	Farming	43 (30.7 %)	70 (44.6 %)		1.00 (reference category)		
HH monthly inc.	Below Ksh.4,500	60 (42.9 %)	99 (53.5 %)		1.00 (reference category)		
	Ksh.4500-9,000	55 (39.3 %)	42 (26.8 %)	0.002	0.451	0.269-0.754	0.002
	Over Ksh. 9,000	25 (17.9 %)	16 (10.2 %)		0.340	0.167-0.694	0.003
Ability to read	Able to read	114 (81.4 %)	90 (57.3 %)		0.285	0.167-0.486	<0.01
	Unable to read	24 (18.6 %)	67 (42.7 %)	<0.01	1.00 (reference category)		

Legend: HH-Household, ACT- Artemisinin-based combined therapies, inc.**-**Income. From the results above, education level, household monthly income and ability to read affected the proportions of those that adhered to ACT. The *P-*values in bold are statistically significant at *P ≤* 0.050. ^a^Statistical significance determined by Chi-square tests. ^b^Statistical significance determined by logistic regression tests.

### Factors associated with ACT adherence

In order to determine factors associated with adherence to ACT in Southeast Alego location, multivariate logistic regression analyses were performed. The independent variables included demographic (age, gender, marital status, household head, and household size), socio-economic (household source of income, household monthly income, education level and ability to read) and environmental factors (source of drugs, distance to source of drugs, and availability at source). The above factors were regressed against the outcome variable; adherence. The full regression model of the demographic factors revealed that age was a significant factor in determining ACT adherence since many respondents (57.9%) who were <13 years (OR, 0.571; 95%CI 0.306-0.905; *P =* 0.017) adhered as opposed to respondents who were ≥13 years (42.1%) (Table 3). Consistently, logistic regression model demonstrated that low age (OR, 0.571, 95% CI, 0.360-0.905; *P* = 0.017), higher education level (OR, 0.074; 95% CI 0.017-0.322; *P* < 0.01), ability to read (OR, 0.285, 95% CI, 0.167-0.486; *P* < 0.01) and higher income (Ksh. > 9000; OR, 0.340; 95% CI, 0.167-0.694; *P* = 0.003) were associated with ACT adherence (Tables 3–5). However, adherence was not significantly associated with gender, HH size, HH head, marital status, source of ACT and distance to the source (Tables 3–5).

**Table 5:** Environmental factors associated with adherence to ACT in Southeast Alego location

**Independent variables**	**Adhered**	**Did not adhere**	**P-value**^**a**^	**OR**	**95 % CI**	**P-value**^**b**^
Environmental factors						
Source of ACT						
Health facility	132 (95.7 %)	144 (91.7 %)	0.162	1.00 *(reference category)*		
Bought drugs from Chemist	2 (1.4 %)	1 (0.6 %)		0.458	0.041**-**5.114	0.526
Left-over, given by neighbour /friend /relative	4 (2.9 %)	12 (7.6 %)		2.750	0.866**-**8.737	0.086
Availability at source						
No	60 (42.9 %)	97 (61.8 %)	0.001	1.00 *(reference category)*		
Yes	80 (57.1 %)	60 (38.2 %)		0.464	0.292**-**0.738	0.001
Distance to source						
≤ 30 minutes	67 (47.9 %)	66 (42.0 %)	0.350	1.00 *(reference category)*		
> 30 minutes	73 (52.1 %)	91 (58.0 %)		1.265	0.800**-**2.002	0.314

Legend: HH- Household; ACT- Artemisinin-based Combination Therapy. The results above show that availability of ACT at the source was significantly associated with adherence to the drug and it was not associated to the source and distance to source of ACT. *P*-value in bold is statistically significant at *P ≤* 0.050. ^a^Statistical significance determined by Chi-square tests. ^b^Statistical significance determined by logistic regression tests.

### Accessibility of ACT

Accessibility was assessed by distance to drug source, drug availability at the source, affordability and acceptability of ACT. Assessment of distance from the drug source revealed that majority of the respondents cover longer distances (55.2%) to reach the source of ACT. Moreover, 52.9% of the respondents reported that ACT was not always available at the source. Further analyses revealed that drug availability (*P =* 0.020) and distance to drug source (*P* < 0.01) significantly varied in the sub-locations (Table 6).

**Table 6:** ACT availability and distance to source per sub-location

	**Availability at source**			**Distance to source**		
Sub-location	Yes	No	P‐value	≤ 30 minutes	>30 minutes	P‐value
Masumbi	45 (55.6 %)	36 (44.4 %)		27 (33.3 %)	81 (66.7 %)	
Nyang’oma Kogelo	17 (31.5 %)	37 (68.5 %)		34 (63.0 %)	20 (37.0 %)	
Mur Ng’iya	31 (41.9 %)	43 (58.1 %)	0.020	26 (35.1 %)	48 (64.9 %)	0.001
Bar Agulu	47 (53.4 %)	41 (46.6 %)		46 (52.3 %)	42 (47.7 %)	
Total	140 (47.1 %)	157 (52.9 %)		133 (44.8 %)	191 (55.2 %)	

Legend: From the above results, distance to source and availability at source was significantly different across the sub-locations. ^a^Statistical significance determined by Chi-square tests. *P-*value is significant at *P ≤* 0.050.

In order to determine affordability, Key Informants were asked about the cost of malaria treatment at the health facilities. The respondents were also asked to comment on ACT treatment. The results revealed that ACT was more affordable at government health institutions as compared to other sources. In these facilities, with an average amount of KShs. 40, the respondents could get ACT and analgesics. “What complicates this is the distance to these health facilities”, noted one of the key informants. At the dispensing chemist, ACT was sold at KShs. 250 and KShs. 450 (for child and adult dose, respectively). In addition, the dispensing chemist operators reported that as a result of the high costs, they do not stock a lot of ACT, since they end up expiring on the shelves. However, there were ACT (generics) which retail at KShs. 40 in one chemist, and these were affordable to a majority of the study population.

In addition, acceptability level to ACT in treatment of malaria was also examined with reference to the respondents’ perception on its usage. The results revealed that acceptability was high (95.4%) since only 4.4% of those who used ACT were not content with it. Others complained of the number of tablets taken (28.7%), duration of treatment (9.5%) and the smell, taste and color (29.1%) to be discouraging, however, they said that when taken, it cures malaria (Table 7).

**Table 7:** Respondents perception on ACT use in treatment of malaria

**Perception**	**Frequency**	**Percentage (%)**
ACT (Coartem) treats malaria	45	15.2
ACT (Coartem) do not treat malaria	13	4.4
People adhere to ACT (Coartem prescription)	29	9.8
Duration of treatment is too long hence non-adherence	28	9.5
ACT tablets are too many hence non-adherence	85	28.7
ACT (Coartem) has bad taste, smell and color	86	29.1
People do not adhere since when they start feeling better, they forget and discontinue treatment	10	3.4

Legend: Most of the respondents reported that ACT had bad taste, smell and color (29.1 %) and numbers of ACT tablets taken were too many (28.7 %) but it cures malaria. Only 4.4 % were not content with the drug.

## Discussion

It is of great importance to understand malaria treatment through the use of ACT and its adherence with the main aim of improving the usage patterns of this valuable drug. Adherence to drug prescription is a key factor in determining drug efficacy and effectiveness. Non-adherence to prescription can translate to resistance by the disease causative agent [21]. The current study was designed to investigate adherence level, factors that influence adherence and accessibility to ACT in western Kenya. The findings from this study demonstrated that non-adherence level was high (53%) in this region. The results further revealed that most people took more or less than 3 days to complete their ACT treatment. In addition, individuals did not take the recommended number of tablets even if they took them within the recommended duration. This observation is consistent with findings of other studies in Cambodia and Maheba in Zambia in which high non-adherence to ACT, SP and artesunate were reported [21,22].

Age, education level, household monthly income and ability to read significantly varied between the adherent and non-adherent respondents. Individuals with formal education who were able to read the prescriptions were likely to adhere to ACT. In addition, majority of those earning moderate household monthly income, which in essence translate to ability to purchase the ACT at the recommended dosage also were adherent. These observations were supported by regression analyses that identified age, education level, ability to read, household source of income, household monthly income and availability of drug (ACT) at source to be significantly associated with ACT adherence. On the household source of income, it was demonstrated that families which had salaries and who were self employed adhered more compared to those that relied on farming. This could be attributed to the fact that, the salaried respondents had some level of formal education and could therefore read the prescriptions. Economically, farming in this area was less productive and this could also limit the ability of the households to purchase ACT if unavailable from the government health facilities. The results presented here, however, show that gender, household head, household size and marital status did not influence adherence to ACT prescription. Consistently, a previous study carried out in Turkey to determine knowledge on malaria and behaviour in an endemic rural area demonstrated no relationships between adherence and race, gender, educational experience, household head and marital status [23]. However, the results of the current study are inconsistent with a previous study in Sudan among college students examining self-medication practices with anti-biotics and anti-malarials. In this study, gender was found to be a significant factor in determining anti-malarial and antibiotic use. In the same study [24], as well as another community-based cross-sectional survey in the Tulu district in southern–central Ethiopia [25], age was a significant factor in determining treatment-seeking behaviour for malaria. The lack of influence of gender, household head and marital status on adherence to ACT could be attributed to the fact that once one acquire malaria, the preceding conditions irrespective of the listed status would call for immediate medication.

A further assessment of socio-economic factors that could influence adherence revealed that household monthly income, education level and ability to read influenced adherence. This is because majority of households with income of more than KShs. 9,000 ($112) per month took correct dosages as opposed to those with income of below KShs. 4,500 ($56) or between KShs. 4,500-9,000 ($56-112). These results are comparable with a study in Tanzania in which it was demonstrated that children from relatively poorer households were less likely to be adherent to prescription as compared to those from wealthier families [26]. In the current study, it can as well be argued that higher income households are able to afford buying drugs when required. Consistently, a study in Maheba refugee camp in Zambia, reported that the refugees were leaving in precarious conditions and this affected their socio-economic conditions possibly resulting to high non-adherence [21].

Assessment of environmental factors that can influence drug adherence revealed that availability at source influenced pattern of adherence. Availability of drugs determines which drugs are obtainable for use at the source. Majority of the respondents mainly relied on government health institutions as source of these drugs. This is because most of the respondents were relatively poor and could not afford to buy these drugs at the retail pharmacies or visit a private clinic. Government health facilities are not reliable sources of the drug since most of them suffer ‘a drug is out of stock syndrome’ and this could be a possible reason for non-adherence. However, when given at these government health institutions, they are given full dose unlike when they buy the drugs independently. These findings are, however, inconsistent with those of a study carried out in southern Ethiopia on self-treatment of malaria in rural communities, where anti-malarial drugs were bought from markets or any shops because it was close to home (43.6%), the drugs were available (25.8%), the shops were easily accessible (20.9%), and waiting time was short (11.5%) [27]. Moreover, it is worth noting that distance to the source of drugs in the current study did not influence adherence in this study. Previous studies have shown that household distance to health facilities is a critical determining factor to adherence. For example, the study in Maheba, Zambia established that the refugees while fleeing from war and violence, were surviving on limited resources for many years, and did not have regular access to health care or education; hence, this affected their adherence to ACT prescription [21].

Assessment of accessibility of ACT revealed that availability of the drug was low since ACT was not always available at its source and majority covered longer distance to the source. These results are comparable to previous ones in Cambodia in which access to ACT in remote areas was determined and shown to be low [28]. Another study conducted in Kenya to assess access to anti-malarials revealed that majority of ill individuals, especially the poor, had no access to prompt effective treatment [29]. Additional studies on policy in practice in Kenya showed that health facilities suffered from chronic drug shortages due to delays in drugs deliveries from the central level and the failure to adjust drug quantities to suit seasonal fluctuations in disease burden [30].

In addition, the current results revealed that ACT is affordable at government health institutions. Patients aged 5 years and below are treated free of charge and those above 5 years are charged an average of KShs. 40, after which they are issued with Artemesinin Lumefantrine (AL) and analgesics. Most respondents cannot purchase the same drugs in pharmacy outlets since the prices are KShs. 250 (~$4) as compared to the KShs. 40 ($0.5) in government health institutions. The issue on high costs could also explain the reason why most of the respondents reported that when ACT is not available at the health facility, they buy cheaper alternative anti-malarials or just analgesics and wait until ACT are available. Other studies have also revealed that reduced charges at health facilities can improve accessibility [29,31,32].

Furthermore, ACT acceptability in treatment of malaria was high in this study since only 4.4% of those who used ACT said that they were not content with its usage. Majority reported that ACT was effective in treatment of malaria with a small proportion believing that it was not effective. The results are consistent with those of clinical trials on ACT in the treatment of multi-drug resistant and acute *P. falciparum* malaria, which demonstrated favourable tolerability, high efficacy and adequate parasitological response to ACT in general and in particular to the standard 6-dose regimen [33,34]. The results of this study also revealed that there is inadequacy in the supply of ACT in the study area. Most of the health institution in this area continually battle with ‘drug is out-of-stock syndrome’. In Kenya, adequacy of supplies of these drugs is a challenge. Often, public health facilities do not have the recommended anti-malarials in stock. Lack of medicine in the government health facilities contributes to people buying drugs over-the-counter (OTC), where the quality of the drugs is not controlled and information on dosage is not often provided [30].

### Study limitations

The study was based on both self-reported and medically-diagnosed episodes of malaria experienced in the previous two weeks prior to the interview. Hence, we could not verify the authenticity of the information obtained from the respondents. Secondly, the case-specific definition of malaria could not be ascertained in this study, since some sought to over-the-counter drugs when they had symptoms similar to those of malaria. Moreover, there was recall bias in this report. Participants were asked questions based on a two-week recall period. Some of the participants would therefore, not vividly remember whether they actually had malaria/fever or how they took the drugs in the last two weeks preceding the interview. To increase accuracy on duration of treatment, hospital cards were reviewed and the drug sachets observed and number of tablets remaining or taken were counted. This certainly affected the quality of this study. However, the two week recall period is the reference period used by WHO surveys and has been adopted by many researchers for similar surveys. Therefore, findings in this report are of comparable quality. Similarly, structured interview-based studies are limited by the pre-determined responses, which are susceptible to response bias. However, attempts were made to minimize this potential source of bias by testing the tool, training field assistants in its administration, and amending it to make the wording familiar and culturally-appropriate. Finally, not asking study participants how they took Co-Artem or what advice they were given about how to take their Co-Artem was the other major limitation of the study since we could not asses the level of failure rates as a result of these components. This follows previous observations that absorption of hydrophobic lipophilic lumefantrine is greatly increased by a small amount of fat (1.2 g) co-administration [35] and failure to implement this component during prescribing result in increased failure rates [36].

## Conclusions

This study has shown that the key challenge in changing malaria treatment policy to ACT is patients’ adherence to drug prescription and ensuring adequate access to ACT in poor rural settings where communities have limited health care services. Adherence level was low in the study area and there is potential need for interventions to improve patient adherence. Patient-centered care will be vital to encourage adherence to new treatment algorithms developed in response to the changing malaria environment in Kenya. Behaviour-change-communication (BCC) programmes should be initiated to aid in awareness creation on the consequence of non-adherence [37-39]. Monitoring patient adherence is thus quite necessary to maintain ACT efficacy. There is also need for equitable distribution of resources in the country to ensure that the remote rural areas have access to quality formal education and drugs since these significantly influence accessibility. In addition, through Affordable Medicines Facility for Malaria (AMFm) funded through Global Fund, it is anticipated that people suffering from malaria will have access to inexpensive and effective anti-malarial treatment. Further studies should be considered on accessibility to ACT when the AMFm funds become available since adherence to the drug regimen may be influenced by several factors ranging from policy to demand and supply. Future studies should mainly assess these policy factors.

## Competing interests

The authors declare that they have no competing interests.

## Authors' contribution

OEO, GA, ER and CO designed, carried out the survey studies in the rural population and participated in the drafting of the manuscript. GOO, WO, RW, CAW, TW, JMO, SG, and SBA performed the statistical analyses and participated in the drafting of the manuscript. All authors read and approved the final manuscript.

## Author’s information

Department of Public Health, Maseno University, Maseno, Kenya (CAW); University of Minnesota, Centre for Global Health Research, Kenya Medical Research Institute, Kisumu, Kenya (GA); Senior Lecturer in Department of Pathology, Kenyatta University, Nairobi, Kenya (TW); Senior Lecturer in Department of Biochemistry and Biotechnology, Kenyatta University, Nairobi, Kenya (GOO); Senior Lecturer at the Department of Nursing, Masinde Muliro University of Science and Technology (JMO); Associate Professor and Dean, Faculty of Arts and Social Sciences (FASS), Maseno University, Maseno, Kenya (SG); Lecturer, Department of Biochemistry, Maseno University, Nairobi, Kenya (SBA and WO); Doctoral student at the Department of Biochemistry and Biotechnology, Kenyatta University, Nairobi, Kenya (ER); Director, School of Public Health and Community Development, Maseno University, Maseno, Kenya (CO).

There is no conflict of interest for any of the authors of the manuscript due to commercial or other affiliations. The study was approved by the ethical and scientific review committees at Maseno University.

## Pre-publication history

The pre-publication history for this paper can be accessed here:

http://www.biomedcentral.com/1471-2334/12/143/prepub
